# Necessity of Identifying the Pulmonary Hemorrhagic Spirochetosis (Pseudo Tuberculosis)

**Published:** 1918-11

**Authors:** 


**Necessity of Identifying the Pulmonary Hemorrhagic Spirochetosis (Pseudo Tuberculosis).** By Dr. F. BARBARY. *Bulletin de l'Académie de Médecine*, June, 1918.

Since the report of MM. Louis Martin and Auguste Pettit in October 1916, on ictero hemorrhagic spirochetosis, which was followed by an account by Professeur Chauffard of the research work of the Japanese, English and French authors, attention has been attracted by the spirochetic affections.

In July, 1917, Doctors Cristau and Plazy studied at Lorient a great number of patients with polymorphic symptomatology, and they gave a note on " the hemorrhagic spirochetosis at Lorient ".

Dr. Pettit, having examined the Lorient patients, said that Lorient spirochetosis was different from the ictero-hemorrhagic spirochetosis. His affirmation was based on the following facts :

1. Urine.

*a*) Abundant quantity of spirochetes in the urine in the Lorient spirochetosis.

*b*) Necessity of centrifuging considerable quantities of liquid and of adding culots with the ictero hemorrhagic spirochetosis.

2) Inoculation : immunity of the guinea pig to the Lorient spirochete, but receptivity for the ictero hemorrhagic.

Clinical facts : habitual icterus in the ictero hemorrhagic spirochetosis ; rare in the Lorient spirochetosis.

Forms quite unknown in the ictero hemorrhagic, but revealed in the Lorient spirochetosis; rheumatism, knotty erythema, pleuropneumonia, etc.

Verneo and Briemer attribute trench fever to a spirochete which Jouvry and Dujarrie de la Riviere have observed in a French soldier.

In the course of the various medical examinations made, we have been struck by a great number of elements with flexuous forms in S or C, of quite variable size and with more or less numerous spires.

Once the patients had washed their mouths thoroughly, we collected the sputum in the sterilized boxes of Vietri. In all the phenic acid thionin preparations we have identified a considerable number of spirochetes scattered in epithelic debris, and a reticulum enclosing polynuclear pneumococci.

*Conclusions* : At the present time the search for spirochetes in sputums, urine and blood must complete the bacteriological examinations.

For a long time mycoses have been neglected. Actinomycosis, sporotrichosis, oosporoses, now investigated in like aspergillosis, have cured various affections attributed to syphilis or tuberculosis.

It is probable that a number of pathological states, imperfectly known, now belong to spirochetosis. When the time of sorting the various forms of tuberculosis takes place, in view of the reform of treatments in hospitals, a very minute examination must be made and the broncho-pulmonary spirochetosis must be counted among pseudo-tuberculosis

Temperature is at times very slight; auscultation signs are those of bronchitis. Hemorrhage, slight but persistant; bleeding bronchitis sputum; hemoptoical and viscous raspberry colored, are clinical phenomena which will easily attract attention.

Bacteriological examination will reveal at once abundant spirochetes in sputum.

Broncho-pulmonary spirochetosis is extremely contagious, and, apart from direct contagion, the sputum drying up rapidly facilitates the dispersion of the germs.

Sputum receptacles should be half filled with antiseptic liquid. Irrigations of the mouth with oxygenated water and hydrate of chloral should be enforced on the patients in view of so absolutely affirmed a contagion.

